# Pre-hospital care in burn injury

**DOI:** 10.4103/0970-0358.70720

**Published:** 2010-09

**Authors:** Prabhat Shrivastava, Arun Goel

**Affiliations:** Departments of Burns, Plastic, Maxillofacial & Microvascular Surgery, Lok Nayak Hospital & Associated Maulana Azad Medical College, New Delhi – 110 002, India

**Keywords:** Burn injury, first aid, pre-hospital care, triage

## Abstract

The care provided to the victims of burn injury immediately after sustaining burns can largely affect the extent and depth of the wound. Although standard guidelines have been formulated by various burn associations, they are still not well known to public at large in our country. In burn injuries, most often, the bystanders are the first care providers. The swift implementation of the measures described in this article for first aid in thermal, chemical, electrical and inhalational injuries in the practical setting, within minutes of sustaining the burn, plays a vital role and can effectively reduce the morbidity and mortality to a great extent. In case of burn disasters, triage needs to be carried out promptly as per the defined protocols. Proper communication and transport from the scene of the accident to the primary care centre and onto the burn care facility greatly influences the execution of the management plans

## INTRODUCTION

Improved understanding of the pathophysiology and the advances made in burn management over the past 50 years have contributed immensely to the dramatic rise in the survival and reduced morbidity from major burn injuries. The aim has always been to achieve wound healing as early as possible and to minimise the morbidity. The efforts towards this should commence right from the scene of the burn accident, at the time of first interaction between the casualty and the first responder. The care the victim receives within the first few hours after sustaining a burn injury largely determines the final outcome of the management.

## WHO ARE THE LIKELY VICTIMS AND WHY EVERYBODY NEEDS TO KNOW THE FIRST AID?

A burn accident can happen at any place and at any time and medical personnel are usually not around. So, all people should be aware of what the first aid for burns is, and should be able to administer the same, immediately, at the site of the accident. This is most often provided by non-professionals, i.e., friends, relatives, bystanders, etc., at the accident site. In fire accidents, by the time the expert medical help arrives, the injury might become lifethreatening. There is no time to wait for seeking an expert help. The first aid has to be provided by the bystanders, who become the “first responders”. Every second is precious and the quicker the first aid is provided, the minimal is the extent of damage.

## ESSENTIAL INGREDIENTS FOR FIRE

For any fire to happen, three essential ingredients required are oxygen, fuel and ignition. Without these, the burning process cannot begin (or continue). Eliminating any of the three components necessary for combustion will extinguish/prevent the fire.[1] In the case of human body, the body tissues provide the fuel, oxygen is present in plenty in the atmosphere and the ignition is provided by the spark from any source.

## PRE-HOSPITAL CARE

Pre-hospital care or on-site management” implies the management at the site of the trauma and constitutes the major part of the first aid. It begins at the scene of the accident and concludes when specialised/institutional medical care is obtained. It must be readily available, easy to use by the general public, halt the progression of the injury and not hinder professional examination or treatment of the wound at a later date.[2] Promptly administered pre-hospital care, in an effective and systematic manner reduces the extent and limits the depth of burns, thus minimising the morbidity. Here, two important points must be borne in mind:

- We must not get injured ourselves while providing the first aid to the burn casualty.
- Fire fighting is not the job of the person administering the first-aid.

Although the basic principles of first aid remain the same for all categories of burns, the immediate care at the scene of the accident has certain peculiarities for each group.

### Thermal burns

1. *Rescuing the victim from the burning premises:* Should always take precedence over putting out the fire. The victim should be removed from the heat source and moved to a safe place. STOP and DROP “policy” should be followed. Prevent the victim from running which would only fan the flames and make them burn faster. The victim should be instructed to lie down on the floor with the burning side uppermost. As the flames always burn upwards, lying flat not only prevents the flames from involving the face, head and scalp hairs, but also prevents the fire from going around the body.
2. *The casualty should not be rolled on the ground* (as has been practiced traditionally). This manoeuvre can transfer the fire to previously undamaged areas and can also cause other injuries.[1]
3. *If the victim is unable to walk* or is unconscious, as is often seen after smoke inhalation victims entrapped in a closed space, make him/her lie supine on floor with both upper limbs placed extended by the side, above the head and then drag the victim out of the room holding his/her legs.
4. *If there is lot of smoke along with the fire:* The rescuer should tie a rope around his waist so that another bystander can pull him to safety, if needed. Stay low/ crawl on the floor to minimise the inhalation of the toxic fumes. The visibility is comparatively better at the floor level as the smoke, gases and hot air tend to rise. Moreover, breathing should be done through the wet handkerchief to filter out the fumes, carbon and other toxic particles.
5. *Put out the fire in an expeditious manner:* Stopping the burning process is mandatory to prevent further damage.
  - *The flames should be doused with water.* Pour water in copious amounts over the victim to extinguish the flames. The victim or the burnt part can be put under the running tap/shower. Hose-pipes are also handy in these situations. However, the water stream should not be directed over the burnt face. High pressure stream increases the pain and can cause damage to the eyes.
  - Smouldering clothing should be removed.
    - *If water is not available:* Any other non-flammable “clear” liquid such as milk, canned drink can be used.
    - The victim should be put on the ground with the burning side uppermost and then wrapped in a heavy cotton cloth (blanket/rug/*dari*/coat or any other heavy fabric). *Nylon or other inflammable cloth material should not be used for this purpose*. Covering the burning body excludes the air, thus depriving the fuel (body tissues) of its oxygen and the fire extinguishes. Once that is done, the blanket should be immediately removed as it tends to retain the heat.
    - Fire extinguishers are extremely useful in putting out the fire.
    - Don’t throw/apply mud/sand over the victim’s body to put out the fire.
6. Make the victim lie supine. Watch for the response and assess for ABC (Airway, Breathing, Circulation). If there is no response and there are no chest movements, cardiopulmonary resuscitation (CPR) should be instituted urgently.
7. *Cooling the burn:* The first objective in the burn wound care is to dissipate the heat. The subcutaneous temperature continues to rise for a while even after the heat source has been removed. Thereafter, it takes about 3 minutes for the tissues to return to body temperature.[3] Immediate active cooling of burn wounds with cool tap water (lavage, soaks, compress or immersion) is effective. Continuous cooling for the first 10 minutes dissipates heat, reduces pain, delays onset and minimises the extent of burn oedema by decreasing the histamine release from the skin mast cells.[4,5]
  - Cool soaks aid in the cooling process only for the first 10 minutes following the burn injury. After this, they no longer assist in cooling the burn, but may provide pain relief to the patient.[6]
  - However, in infants, young children and in adults with > 25% total body surface area (TBSA) burned, prolonged irrigation/cooling can lead to hypothermia. Cooling process should not cause shivering. Significant drop in body temperature is associated with ventricular fibrillation.
  - Application of ice/ice-cold water may lead to numbness and intense vasoconstriction, causing further damage to the tissues. Hypothermia is also a major risk with ice-cold water.
  - Cooling of the burn wound should be done at the site of the incident as by the time most patients present for care, the burnt tissues have already cooled spontaneously maximising the damage.
8. *Once the fire has been extinguished: All* the burnt clothes (including belts, socks and shoes) should be carefully removed from the victim’s body. Fabric that has melted and is *stuck* to the burn wound should be left in place. The ornaments (necklace, wrist watch, bangles, bracelets, nose-rings, ear-rings, rings around the fingers and toes, anklets, etc.) should also be removed as they retain heat and continue tissue damage for a prolonged period. Rings around the fingers and toes can cause constrictive tourniquetlike effect, severely compromising the circulation to the distal portion of the digits, once the oedema sets in.
9. *Do not break any blisters:* Management of the burns blisters has been controversial. Opinions vary from immediate removal,[7] delayed removal[8] or leaving them intact.[9] The decision to de-roof/puncture/ aspirate the blisters should be left to the burn specialist.
10. *Do not apply any medications locally:* Do not apply any ointments, creams, lotions, powders, grease, *ghee*, gentian violet, calamine lotion, toothpastes, butter, colouring and other sticky agents, etc., over the burn wound. They make the formal assessment of the nature, depth and extent of the burn wound difficult.
  Moreover, removal of such substances might also be difficult and painful to the patient.
11. *Prevent contamination:* The burnt part should be wrapped in a clean, dry sheet/cloth. Pillow cover/ plastic bags are suitable for upper and lower limb injuries. Plasticised polyvinyl-chloride (PVC) film available as a food-wrap is a good alternative to cover the burned areas.[10] Being pliable, it moulds to the contours of the wound and forms an impermeable, non-adherent barrier. Its application and removal is easy and painless. Moreover, being transparent, it also permits inspection of the wound. Wrapping the burn wound
  - Minimises contamination by shielding the burn wound from secondary infection,
  - Reduces pain produced by the exposure of the damaged nerve endings (in partial thickness burns) to the air currents and
  - Provides protection during transport.
12. *Recognition of the associated injuries:* Chaos, panic and attempt to escape from the incident site can, besides the burn trauma, result in injuries to head, spine, upper and lower extremity, chest and abdomen. Fractures should be immobilised/splinted and the bleeding controlled by compression. Patients with suspected injury to head and spine should not be moved much as this could worsen the damage to the spinal cord.
13. *Providing pain relief and reassurance to the patient:* Extreme physical pain because of the exposure of the free nerve endings in a partial thickness burn injury and the emotional trauma generated by the circumstances are the twin challenges that need our immediate attention.. Prompt and continuous cooling of the burnt areas for 10 minutes and covering it with a sterile, clean, dry cloth help a lot in providing analgesia. Formal oral analgesics are usually not needed in the first aid. Reassurance and consolation to the victim and the family are important components of early care.
14. Other measures
  - *Seek immediate medical help:* While the first aid is being administered to the burn victim, call for the paramedical/medical help which, if arrives in time, can be invaluable, especially if the victim goes in shock. It is always better if medical or paramedical personnel accompany the victim to the hospital.
  - *Withhold oral intake:* It is advisable not to give the victim anything to drink / eat as this may result in vomiting, especially if the TBSA burnt is >25%.
  - Victims with additional component of inhalation injury are comfortable in sitting position. They should be given 100% oxygen, if available, during transport.
  - Advanced paramedical procedures like intravenous infusion and endotracheal intubation are usually not required at the scene of burn accident.[1]
  - *Co-morbid conditions/other pre-existing illnesses:* Comorbidities always influence burn care and should be enquired. Victims of alcoholism and drug addiction need special attention. as do pregnant ladies and lactating mothers.
  - If the victim is drowsy/unconscious, possibility of inhalation injury, head injury, chest trauma, abdominal trauma, shock should be kept in mind.

#### Arrange transport to the medical facility

- While the first aid is being provided to the victim(s), always call to arrange for the transport to the nearest hospital. While air ambulances are a rarity, efficient surface transport with basic life support systems are essential.
- The victim(s) should be transported directly to the burn centre, if the centre is located within 15 minutes distance from the site of the accident.
- However, if there is no burn centre in the vicinity, the victim(s) should be taken to the nearest hospital where the formal assessment of the percentage of the burns, associated injuries and the resuscitation shall be done. This may include intravenous line for administering fluids, catheterisation of the urinary bladder, insertion of the nasogastric tube, oxygen administration by mask, endotracheal intubation, etc., as per the condition of the patient. Circumferential burns over extremities, digits, chest, abdomen, neck should be carefully assessed. These may require escharotomy during resuscitation to prevent circulatory and/or respiratory compromise.
- Secondary transportation to the burns centre should be properly arranged in a less hurried manner, with a stabilised patient accompanied by a qualified physician/nurse.
- The intravenous fluids and oxygen should be administered even during the transport. Adequate analgesia should be ensured prior to the transfer. Enough IV fluids, emergency drugs, oxygen cylinder, ambu-bag, blankets, etc., should be available during the transport.
- *Inform the burn centre:* Before commencing the journey, it is better to communicate to the burn centre about the number of casualties, their condition and the approximate time of arrival.
- Records of the fluids and other medicines administered should be handed over to the medical personnel accompanying the victim(s) during the transfer.
- Do not panic and do not drive recklessly to the hospital.

### Chemical burns

1. Chemical burn injuries are caused by contact, ingestion, inhalation of noxious fumes of acids, alkalis or organic materials. All chemical burns are classified as major burn injuries and preferably be transferred to a burn centre.
2. Almost 25,000 chemicals have been identified to cause chemical burns. The commonest are due to alkalis and inorganic acids. If the solution in which the chemical is contained is hot, the resultant injury is a mixture of thermal and chemical burns.
3. The process of tissue damage in chemical burns stops only when the chemical is either neutralised by the tissues/antidote or is sufficiently diluted/washed away by irrigation with water. The alkali burn injury is frequently deeper than it initially appears.
4. Acids, e.g., sulphuric acid, hydrochloric acid, etc., produce a coagulative necrosis similar to that of thermal injury. While alkalis, e.g., sodium hydroxide produce a colliquative or liquefaction necrosis, burrowing deeply into tissues with an effect that can last for several hours.[11] Cement is a common cause of alkali burns.

#### Guidelines for first aid for chemical burns:

- Rescuer must wear protective gloves, mask, eye protectors, etc., to avoid coming in contact with the chemical. They should not become a victim themselves by contamination.
- Clothing/ornaments/watch/belt/socks/shoes, etc., impregnated and contaminated with chemicals, should be immediately and completely removed since external irrigation is ineffective.
- Thorough irrigation of the affected areas with copious amounts of water significantly reduces the size and severity of the injury.[12,13] Irrigation should be done by keeping the affected area under running tap water/shower to neutralise/flush away the noxious chemical. It should continue for 2 hours in case of acid burns and for at least 12 hours in case of alkali burns.
- Water is contraindicated as a first aid measure in chemical burns caused by the heavy metals like sodium, potassium and calcium. They react violently and explosively with water to produce caustic hydroxide liberating much heat in its production and thus result in combination of thermal and chemical burn. Immediate treatment in these cases is to brush off/pick out from the skin as many particles of sodium or potassium as possible and then to direct a high pressure jet of water at the remainder. Ignition of particles will occur, but if the flow is great enough, the heat will be dissipated by water. Covering the remaining particles with oil, although prevents combustion, cannot halt the tissue damage as the remaining metal particles continue to react with tissue water.
- Time should not be wasted in looking for the specific antidotes.
- Speed in irrigation is also important as certain organic solvents are quickly absorbed into the blood stream via the skin or by inhalation and cause systemic toxicity.
- Irrigation should continue even during the transport to the hospital.
- Never apply acid to base, or base to acid as it can cause exothermic reaction generating heat resulting in further damage.
- Litmus paper, if available, can be used to confirm complete removal of acid or alkali.
- Accidentally swallowed caustic chemicals should not be treated by inducing vomiting. Instead, milk should be given to drink to act as a buffer.
- Victims of mass casualty due to contact with the hazardous materials (Hazmat) should be removed from the zone of immediate danger and then decontaminated. Decontamination at a hospital is discouraged due to potential spread of the substance to other patients. All the areas utilised for decontaminating victims must themselves be decontaminated after use.[14,15]

#### First aid for chemical burns of the eye/cornea

- Chemical burns of the eye are potentially serious (specifically alkali burns) as it can cause corneal ulceration and even loss of vision. The chemicals lodge in the upper and lower conjunctival fornices and are not easily washed away.
- Immediate copious irrigation of the involved eye with normal saline or water should be commenced without looking for the specific antidote(s). Eyelids should be widely separated manually to allow flushing away of the chemical from the fornices and the medial and lateral canthi. Due to severe pain and blepharospasm, it may be necessary to hold the eyelids open by a second person. Solid particles of lime need to be removed with forceps.
- Use of high pressure stream of water should be avoided as it may worsen the damage.
- Tilt the head of the victim towards the side of the affected eye to prevent the chemical from entering the canaliculi and nasolacrimal ducts. The chemical agent, even diluted by washing, can cause irritation and inflammation of the mucosa of these ducts, leading to oedema and fibrosis.
- The victim should be seen by an ophthalmologist. The irrigation of the affected eye should be continued during the transport. Once in the hospital, copious irrigation should be continued with normal saline or Ringer lactate solution. It is recommended to flush the affected eye with a minimum of 2 l over 30 minutes to 1 hour and continue until the normal pH (7.4) of the eye surface has been restored.[16]
- If a victim has combined facial and eye burns, the eye takes the precedence and the victim should be first seen by an ophthalmologist and later by the burn surgeon.

### Coal tar/bitumen burns

1. Coal tar is used to resurface the roads and roofs in its molten form. On contact with the skin in the molten state, it actually causes contact burns (thermal injury). It does not produce a chemical injury. The molten tar adheres strongly with the epidermis. Rapid cooling of the hot adherent material with the cold running water (for 30 minutes), as the first aid measure, limits the damage to the skin.[17] Initial heat of contact sterilises the skin and the tar acts as a dressing of the burn wound.
2. It is not necessary to remove the adhered tar (unless eyes are burned) as any such attempt leads to further damage to the skin. Rarely, when removal of the tar is contemplated, gentle wiping with the swabs soaked in the cooking oil/ fat is safe but tedious procedure.[11]
3. Although tar is soluble in kerosene, petrol and other petroleum based solvents, they should not be used to remove the adherent tar since they may cause further tissue injury and even systemic toxic effects.
4. After cooling, material adherent to blisters can be removed when the blisters are debrided, but that adherent to the unblistered tissue should be covered with a ointment and dressed. Daily reapplication of the ointment causes emulsification and atraumatic removal of the tar within 2–3 weeks. Sometimes, full-thickness burns are produced which should be treated as any other thermal burn.

### Electrical burns

Electrical burns occur due to contact with live electric wires or lightning. These injuries are classified under major burns and the victim needs to be transported to a burns care facility. Tetanic contractions induced in the muscle prevent the victim to free himself/ herself from the source. Strong contractions of flexor muscle group in upper limb tend to maintain the contact and it may cause muscle injury, joint dislocations and fractures.[18]

Extensive damage to the deeper tissues (muscles, blood vessels, nerves, etc.) may be present beneath intact and unburned skin. Dry skin offers more resistance, thus the flow of current to the deeper tissues is retarded, but the extent of the cutaneous burn is greater. Conversely, as the moist skin offers low resistance, a less significant thermal burn injury manifests on the skin, but current flows more freely through the deeper tissues.

Thermal injuries may also be present due to ignition of the clothing after electric contact. Fractures of long bones and spine may be associated due to fall. Asystole occurs with high voltage contact burns. Cardiac arrythmias and ECG changes are usually seen within first few hours post injury (present in about one-third of patients).[15,19] The involved extremities should be monitored for pain, pallor, paraesthesia, paralysis and pulselessness, which indicate the development of compartment syndrome.

#### Guidelines for first aid for electrical burns

- Do not become a victim yourself.
- Turn off the source of electric supply. Contact between the victim and the power supply should be broken by switching off the power supply. Even after that, some “residual electric charge” may still remain with large capacitors and condensers. Therefore, a victim should be removed with a non-conducting material like a dry wooden stick/pole/wooden chair. Ideally, the first responder must stand on the dry surface during rescue. No such manoeuvre should be attempted while a person is connected to a high voltage source, as the current is likely to “arc” to the rescuer as he approaches.
- Once the area is safe, check Response, Airway, Breathing and Circulation. If there is no response or respiration, the victim most likely has suffered cardiac arrest and CPR should be started immediately at the scene.
- Call for help immediately.
- Examination for associated injuries (head/chest/ abdomen/limb injuries), especially if there has been a fall from a height from an electric pole/rooftop etc. Cervical spine and other suspected fracture sites should be adequately splinted.
- If the victim is conscious, the first aid and the transportation should proceed on the same lines as described for thermal burns.

### Lightning injuries

1. Lightning is a natural atmospheric electrical discharge that occurs between regions of net positive and net negative electric charges. Lightning burns are often superficial and present with a spidery/arborescent pattern markings which rapidly disappear. Burns in the region of the metallic objects like necklace, watches, rings, etc., may also be seen.[20]
2. Cardiopulmonary arrest is the most common cause of death in lightning victims. Three to five times more people survive being struck by lightning each year than those who die, despite the high likelihood of cardiac arrest.[21]
3. The victims of lightning injuries are managed in the same way as those of electrical injuries. First aid involves assessment of the level of consciousness and instituting immediate CPR at the scene if there is no response. *Immediate post injury coma and neurological deficits are common, but they often clear in a few hours or days.* It is important to know that prolonged resuscitation with cardiac massage and ventilation is required. These patients can withstand apnoea for very long periods of time; probably because the lightning injury halts cellular metabolism.[22] Fixed dilated pupils should not be regarded as the sign of death!
4. During thunderstorms, one should remain inside a closed car or in a building away from doors and windows, metal objects such as pipes, sinks, radiators, and plugged-in electrical appliances. When outside and unable to find shelter, it is important to maintain distance from tall trees. Lightning can travel through water, thus it is important to avoid swimming, boating and bathing during a thunderstorm.

### Inhalation burns

1. An inhalation injury (chemical tracheobronchitis and acute pneumonitis) is caused by inhalation of smoke and other irritative products of incomplete combustion. Available oxygen is rapidly consumed by a fire in a closed space so that the combustion is incomplete.
2. Inhalation injury should be suspected in any patient with history of being burned/trapped in an enclosed smoke filled space. The suggestive features include head and neck burns, singeing of nasal vibrissae, agitation/anxiety, hoarseness of voice, stridor/wheeze, dyspnoea, carbonaceous (sooty) sputum, brassy cough, impaired visual acuity, headache, nausea, vomiting, dizziness, tachypnoea and tachycardia.
3. Three primary facets of inhalation injury are: smoke poisoning, carbon monoxide (CO) poisoning and direct thermal injury to the airway. *Smoke poisoning* results from the inhalation of the byproducts of combustion: noxious chemicals, gases and particulate matter (mainly carbon). The smoke reduces the victims’ capacity to escape by limiting vision and by interfering with correct perception. Incomplete combustion of the organic substances (e.g., wood or coal) results in the formation of CO, which is the principal toxin of concern in the pre-hospital setting. It causes poisoning by blocking the transport of oxygen within the blood.[23–25] Impaired oxygenation results in tissue hypoxia which causes most of the symptoms mentioned above.

#### First aid in smoke inhalation primarily comprises:

- removing the victim from the fire/smoke areab) assessment for Response, Airway, Breathing and Circulation; commence CPR at the scene, if needed,
- calling for help,
- victim should be made propped up and given 100% oxygen as soon as available, even during the transport,
- arranging transfer directly to the burn centre/ burn ICU immediately where fluid resuscitation, endotracheal intubation and proper ventilatory support may need to be provided at the earliest.

## TRIAGE GUIDELINES

In the event of a burn disaster, the initial effort is to get the right patient to the right place at the right time with the right treatment, no matter how limited the resources may be.[26] Sorting out and classifying the causalities to determine the priority, and proper place of treatment and mode of transfer is known as triage. The principle is to identify those victims who are likely to *benefit the most* from the treatment. Factors taken into consideration for triage are the total number of victims, availability of beds and the transportation capability. “*Field triage*” is done by the first responders at the scene of the accident. The “*secondary triage*” from the primary centre to a burn care facility is carried out by the qualified medical personnel.

Griffith (1985) identified the following five groups for victims of burn disasters:[27]

Group I: Minor burns to non-critical sites (<10% TBSA for children, <20% TBSA for adults)
  *Care assigned:* Dressing, tetanus prophylaxis, outpatient care
Group II: Minor burns to critical sites (hands, face and perineum)
  *Care assigned:* Admit, short hospital stay, special wound care, early operations
Group III: 20–60% TBSA burned
  *Care assigned:* Admit to burns unit, IV fluids, careful monitoring
Group IV: Extensive burns (>60% TBSA burned), inhalation injury/associated trauma/illness
  *Care assigned:* Pain medication, psychological support, expectant category
Group V: Minor burns with inhalation injury/associated injury
  *Care assigned:* Administer O_2_, intubation, ventilation, care of associated injuries

The patients in Group I are discharged after first aid or asked to go to nearest primary centre on their own. Group II cases are evacuated at the end. Group III and Group V patients need to be transported first, followed by group IV. Tele-triage can be utilised if available wherein a burn specialist in a tertiary care centre guides in triage and initial management. Initial stabilisation should be obtained at the primary hospital; however, it should be remembered that the burn victims tolerate movement best during the early period following injury, and undue delay may complicate the transfer.[28]
